# Determinants of participation in a post-hospitalization physical exercise program for older adults

**DOI:** 10.1186/s12877-020-01821-3

**Published:** 2020-10-16

**Authors:** Miriam Urquiza, Iñaki Echeverria, Ariadna Besga, María Amasene, Idoia Labayen, Ana Rodriguez-Larrad, Julia Barroso, Mikel Aldamiz, Jon Irazusta

**Affiliations:** 1 Department of Physiology, Faculty of Medicine and Nursing, University of the Basque Country (UPV/EHU), B° Sarriena s/n, 48940 Leioa, Bizkaia Spain; 2 Department of Internal Medicine, Araba University Hospital, BioAraba Research Institute, OSI Araba, University of the Basque Country (UPV/EHU), 01004 Vitoria-Gasteiz, Spain; 3 Department of Medicine, Araba University Hospital, BioAraba Research Institute, OSI Araba, C/ José de Atxotegui, s/n, 01009, Vitoria-Gasteiz, Spain; 4 Department of Pharmacy and Food Science, Faculty of Pharmacy, University of the Basque Country (UPV/EHU), 01004 Vitoria-Gasteiz, Spain; 5 Faculty of Health Science, Public University of Navarra, Navarra, Spain

**Keywords:** Physical exercise, Older people, Participation, Post-hospitalization

## Abstract

**Background:**

Older patients often experience a decline in physical function and cognitive status after hospitalization. Although interventions involving physical exercise are effective in improving functional performance, participation in physical exercise interventions among older individuals is low. We aimed to identify factors that contribute to exercise refusal among post-hospitalized older patients.

**Methods:**

A cross-sectional study of recruitment data from a randomized controlled trial was conducted involving 495 hospitalized people ≥70 years old. Sociodemographic and clinical data were obtained from the Basque Public Health System database. We determined physical function with the Short Physical Performance Battery (SPPB), nutritional status with the Mini-Nutritional Assessment, frailty according to the Fried phenotype criteria, and cognitive function with the Short Portable Mental Status Questionnaire (SPMSQ). Student’s t, Mann-Whitney U, or chi-squared tests were applied for bivariate analysis. Parameters significantly associated with participation were introduced in a logistic multivariate regression model.

**Results:**

Among the analyzed patients, 88.8% declined participation in the physical exercise program. Multivariate regression revealed that older age (OR: 1.13; 95% CI: 1.07–1.19), poor nutritional status (OR: 0.81; 95% CI: 0.69–0.95), and reduced home accessibility (OR: 0.27; 95% CI: 0.08–0.94) were predictors of participation refusal. Moreover, patients who declined participation had worse performance on the SPPB (*P* < 0.05) and its tests of balance, leg strength, and walking speed (*P* < 0.05). No differences were found between groups in other variables.

**Conclusions:**

This study confirms low participation of older adults in a post-hospitalization physical exercise program. Non-participation was associated with increased age, poor nutritional status, and reduced home accessibility. Our findings support the need for intervention design that accounts for these factors to increase older patient participation in beneficial exercise programs.

**Trial registration:**

Australian New Zealand Clinical Trials Registry, ACTRN12619000093189, (date: January 22, 2019, retrospectively registered).

## Background

Older patients tend to experience physical and cognitive decline after hospitalization for an acute illness [1, 2]. Additionally, loss of physical and cognitive capacities may continue months after discharge [3, 4]. Functional decline is directly related to negative outcomes in the year following hospital admission, including dependence, increased risk of institutionalization, and mortality [5]. Total medical expenditures at one year post-discharge grow in proportion to functional impairment, with a potential economic impact due to loss of physical and cognitive function following hospitalization [6].

Physical exercise improves both physical and cognitive performance in older individuals and multicomponent exercise interventions, including resistance, balance, and walking exercises performed during acute hospitalization increase muscle strength, functional capacity, and ability to complete basic daily living activities [7, 8]. Physical exercise also benefits executive function and memory and can reduce the risk of developing dementia [9]. Unfortunately, exercise program participation rates among older adults are usually low [10], including for interventions performed after hospital discharge [11, 12]. Post-hospitalization exercise programs for cardiac rehabilitation or knee or hip repair, in which physical exercise is a well-established part of treatment, have higher participation [13], but most discharged older patients will not participate in such a program. To increase participation in physical exercise programs, it is important to determine factors associated with refusal to participate [11]. Studies exploring older patients’ reasons for non-participation in physical exercise programs have been performed through surveys or interviews without accounting for clinical or functional variables [14, 15]. Thus, the first aim of the present study was to identify reasons for older adults rejecting participation in a physical exercise program after hospital discharge. The second aims was to analyze physical, clinical, and sociodemographic factors associated with non-participation. Understanding the determining factors of exercise refusal will help inform the development of future strategies aimed at increasing older adult participation in physical exercise programs following hospitalization.

## Methods

### Study design and participants

This cross-sectional study was a secondary analysis based on data obtained from recruitment for a randomized controlled trial (RCT) comparing two supervised physical exercise interventions of different lengths of the supervised part [16]. The protocol was registered retrospectively under the Australian and New Zealand Clinical Trials Registry with the identifier ACTRN12619000093189 (date of registration: 22/01/2019). The Clinical Research Ethics Committee University Hospital of Araba (2017–021) approved the study protocol, which complied with the revised ethical guidelines of the Declaration of Helsinki (2013 revision). All participants provided informed written consent before enrollment in the study.

RCT enrollment data were obtained from September 2017 to July 2018 in the Departments of Internal Medicine and Neurology at the Santiago University Hospital of Araba (Basque Country, Spain). Both departments have a high prevalence of older people who usually experience rapid deconditioning due to hospitalization. Eligible participants included men and women ≥70 years who scored ≥20 on the Mini-Mental State Examination (MMSE) [17] and were able to stand and walk independently or with assistance for at least 4 m. The MMSE is a 30-point test used in clinical settings to measure cognitive impairment and to screen for dementia. The cut-off point was set at ≥20 to ensure patients could follow the instructions of the physical exercise program. Exclusion criteria were a diagnosis of chronic kidney disease, autoimmune neuromuscular disease, acute myocardial infarction, or bone fracture in the past three months.

**Fig. 1:**
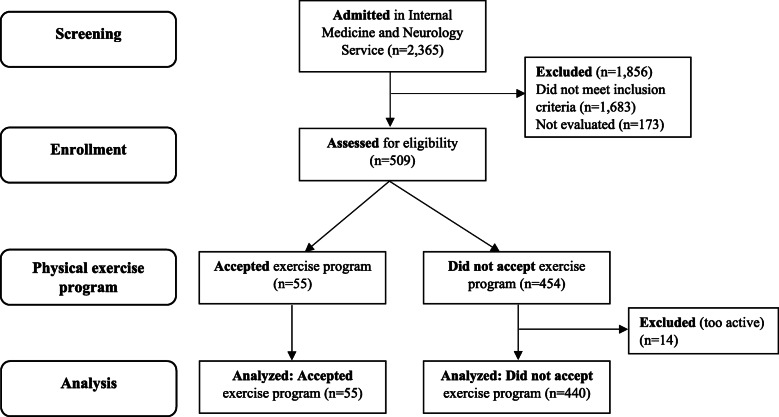
Study flow diagram

During the recruitment period, 2365 non-surgical patients were admitted to the Departments of Internal Medicine and Neurology of University Hospital of Araba, a tertiary teaching hospital in the Basque Country. After screening medical histories and inclusion criteria, 509 patients were eligible to initiate the physical exercise program at discharge. After signing an informed consent document, a comprehensive geriatric assessment was performed by nurses and physicians of the hospital staff and physiotherapists of the research team. Once assessment of each patient was completed, they were given the option of starting a physical exercise program in the same hospital at discharge. Once per week, we also provided informative sessions and distributed informational leaflets throughout the hospital. Of the evaluated patients, 55 agreed to participate and started the physical exercise program, and 454 refused to participate. Those rejecting participation (*n* = 14) in the program because they were active enough on their own were excluded from analysis. Finally, 495 patients were analyzed (Fig. 1).

### Characteristics of the physical exercise program

The characteristics of the program were described previously [16]. Briefly, subjects who agreed to take part in the intervention were randomly assigned to a short (6 weeks) or long (12 weeks) supervised exercise program. In both groups, participants continued the program at home from either 6 weeks or 12 weeks until 24 weeks after the start of the intervention. The program consisted of 1-h group sessions (2 days/week in the supervised part and 5 days/week at home) and included strength, balance and walking training. All sessions began with warm-up exercises (5 min) and continued with strength training of upper and lower limbs (35 min) tailored to the individual’s functional capacity. Balance training exercises (15 min) were also practiced, progressing in difficulty during the program: decreasing arm and base of support and increasing the complexity of movements. Finally, participants were recommended to perform walking sessions on their own. The first participant started the exercise intervention in November 2017 and the last participants ended the program in January 2019.

We asked patients who refused participation in the exercise program to provide reasons for not participating. We categorized refusal reasons as internal reasons, external reasons, and those pertaining to a lack of interest in physical exercise. Internal reasons related to the patients themselves reporting poor health. External reasons related to the patients’ social burdens (travel problems, being a family caregiver, or social problems). Uninterested patients simply had no interest in the physical exercise program.

### Measurements

Sociodemographic and clinical data were retrieved from the Basque Public Health Service’s database. The following data were collected: patient demographic data (sex and age), days of hospitalization, hospitalizations in the previous year, emergency care admissions, and comorbidity (assessed through the Charlson comorbidity index, a method of categorizing patient comorbidities based on the International Classification of Diseases) [18]. We collected the Barthel Index score, which measures performance in basic activities of daily living such as continence and mobility [19]. In addition, we collected the Lawton Index score which measures the instrumental activities of daily living necessary for independence in the community [20]. Finally, we collected socio-demographic data, including patient educational level, whether or not they live alone, and whether they use assistive devices for walking and home accessibility, such as home entrance-related assistance, e.g. an elevator or lift [21].

Physical function was measured individually in patients’ hospital rooms by the Short Physical Performance Battery (SPPB) [22]. SPPB is a battery of tests that combines assessment of balance, gait speed, and lower limb strength. The balance test measured ability to stand for 10 s in side-by-side, semi-tandem, and tandem stands. To assess gait speed, we measured the time to walk 4 m at the patients’ usual speed twice (participants could use a walking aid if necessary). The best walking time of the two measurements was scored. Finally, lower limb strength was measured by time to perform five repeated chair stands, and the sit-to-stand speed was determined. Each test was scored on a scale of 0–4 points, with a total performance score range of 0–12 points using cut-point criteria established by Guralnik et al. [22]. Higher scores indicate better physical function.

Nutritional status was measured using the calf circumference short form of the Mini Nutritional Assessment (MNA-SF) [23]. The MNA-SF is a validated screening tool designed by Nestle to identify elderly persons at risk of malnutrition. The test consists of five questions (appetite or eating problems, recent weight loss, mobility impairment, acute illness/stress, dementia, or depression) and measurement of calf circumference (CC). CC was measured at the calf’s greatest circumference with the patient sitting down, resting their feet on the floor, and knees bent 90°. The test provides a maximum score of 14 points. Higher scores indicate better nutritional status.

Frailty was measured according to the Fried phenotype criteria [24]. A Spanish language version of the frailty performance criteria was used to measure grip strength, walking speed, weight loss, physical activity, and exhaustion.

Cognitive function was measured by the Spanish validated version of the Short Portable Mental Status Questionnaire (SPMSQ) [25]. The SPMSQ includes ten questions to briefly test short- and long-term memory, orientation, and capacity to perform serial mathematical tasks. The total number of errors was counted and one point was subtracted if the patient had a grade school education and one point was added if the patient had a high school education.

### Statistical analyses

Continuous variables were expressed as means with standard deviations (SD), and categorical variables were expressed as frequency counts and percentages (%). Normality of data was assessed using the Kolmogorov-Smirnov test. Sociodemographic characteristics and clinical data of patients who initiated or declined participation in the post-hospitalization physical activity program were compared. Differences in continuous variables were analyzed by the Students’ t test (normal distribution data) or the Mann-Whitney U test (non-normal distribution data), and a chi-squared test was used to compare categorical variables. Variables with *P* < 0.05 in univariate analysis were considered eligible for a backward multivariate logistic regression model to predict participation in the program. The goodness fit of the model was evaluated using the Hosmer-Lemershow test. The Omnibus test was used to determine whether the explained variance was significantly higher than the unexplained variance. Finally, Nagelkerke’s R^2^ estimated the proportion of the dependent variable explained by independent variables introduced in the model. Statistical significance was set at *P* < 0.05. IBM SPSS 21 software (IBM, Chicago, IL) was used to perform statistical analysis.

## Results

This study included 495 hospitalized older adults. The mean age of evaluated patients was 83.6 ± 6.6 years, 257 (51.9%) were men, and the mean Charlson comorbidity index was 6 ± 1.9 points. Of the reasons for hospital admission, 43.6% were infection-related, 27.5% were acute decompensated heart failure, and 13.1% were chronic airflow limitation. The remaining 15.8% of admissions were due to other conditions (falls, delirium, dementia, and others). Almost 9 out of 10 evaluated patients declined participation in a post-hospitalization physical exercise program and 50.7% of patients refused participation due to a lack of interest in the physical exercise program (Table 1).

**Table 1:** Refusal reasons

**Internal reasons**	
Poor health perception	9.5%
**External reasons**	39.8%
Travel problems	24.9%
Other assistance resource	10.2%
Family caregiver	2.6%
Social problems	2.1%
**Not interested in physical exercise program**	50.7%

Patients who declined participation were significantly older than those who initiated the program (*P* = 0.011). Additionally, a significantly higher percentage of patients who needed walking assistance devices (*P* = 0.004) and had poor accessibility at home (*P* = 0.019) did not participate in the program. There were no significant differences between groups regarding sex, education level, living alone, length of hospitalization, or previous year hospital admissions (Table 2).

**Table 2:** Sociodemographic characteristics

	Initiated PE program (*n* = 55)	Declined participation (*n* = 440)	*P*
**Age (years), mean (SD)**	81.7 (5.9)	83.9 (6.7)	0.011^#^
**Sex**			0.874
Men, % (*n*)	52.7% (29)	52% (229)	
Women, % (*n*)	47.3% (26)	48% (211)	
**Education level**			0.563
≤ 12 years, % (*n*)	30.4% (7)	36.4% (122)	
> 12 years, % (*n*)	69.6% (16)	63.6% (213)	
**Length of stay (days), mean (SD)**	6.8 (3.5)	7.6 (4.3)	0.112
**Previous year hospital admission**			0.718
Yes, % (*n*)	36.4% (20)	33.9% (149)	
No, % (*n*)	63.6% (35)	66.1% (219)	
**Previous year emergency care, mean (SD)**	1.7 (2.2)	1.5 (2.5)	0.171
**Walking assistance device**			0.004^$^
Yes, % (*n*)	49.1% (27)	69% (240)	
No, % (*n*)	50.9% (28)	31% (108)	
**Lives alone**			0.346
Yes, % (*n*)	36.4% (20)	30.1% (114)	
No, % (*n*)	63.6% (35)	69.9% (265)	
**Home accessibility**			**0.019**^**$**^
Yes, % (*n*)	94.5% (52)	81.6% (224)	
No, % (*n*)	5.5% (3)	18.4% (50)	

*PE* physical exercise, *SD* standard deviation

^#^ Mann-Whitney U test; ^$^ chi square test

Patients who declined participation had significantly worse functional performance on the SPPB (*P* = 0.015), including poorer muscle strength in the lower limbs (*P* = 0.024), lower balance scores (*P* = 0.011), and slower walking speed (*P =* 0.004), than participants who agreed to the exercise program (Table 3). Further, uninterested patients had lower nutritional status according to the MNA-SF test (*P* < 0.001). Analysis of each component of the test showed that patients who refused to participate in the program had significantly lower scores for both weight (*P* = 0.015) and eating problems (*P* = 0.014). There were no significant differences between groups in Barthel and Lawton scales, cognitive status, comorbidities measured by the Charlson index, or frailty according to the Fried phenotype.

**Table 3:** Clinical, functional, cognitive, and nutritional parameters

	Initiated PE program (*n* = 55)	Declined participation (*n* = 440)	*P*
**Weight, mean kg (SD)**	71.7 (15.2)	67.3 (13.1)	0.021*
**Barthel index, mean (SD)**	87 (17.3)	86.2 (17.5)	0.551
**Lawton scale, mean (SD)**	4.9 (2.7)	4.1 (2.7)	0.065
**SPMSQ, mean (SD)**	1.4 (1.5)	2 (2.1)	0.089
**SPPB, mean (SD)**	6.6 (3.1)	5.6 (3)	0.015^#^
Sit-to-stand speed, stand/s	0.25 (0.16)	0.2 (0.16)	0.024^#^
Balance test	2.9 (1.2)	2.4 (1.4)	0.011^#^
Walking test, s	7.9 (5.1)	11.1 (10.2)	0.004^#^
**MNA-SF, mean (SD)**	11.3 (2.2)	10.1 (2.5)	< 0.001^#^
Eating problems	1.7 (0.5)	1.4 (0.8)	0.014^#^
Weight loss	2.2 (1.1)	1.9 (1.2)	0.015^#^
Mobility	1.9 (0.3)	1.8 (0.4)	0.256
Acute illness/stress	0.8 (0.4)	0.8 (0.4)	0.537
Dementia/depression	2 (0.1)	1.9 (0.3)	0.106
Calf circumference	2.6 (1)	2.3 (1.3)	0.067
**Frailty (Fried criteria), mean (SD)**	2.8 (1.3)	2.9 (1.2)	0.710
**Charlson comorbidity index, mean (SD)**	5.9 (2.1)	6 (1.9)	0.434

*PE* physical exercise, *SD* standard deviation, *SPMSQ* Short Portable Mental Status Questionnaire, *MNA-SF* Mini Nutritional Assessment Short Form, *SPPB* Short Physical Performance Battery

* Student’s t test; ^#^ Mann-Whitney U test

**Fig. 2:**
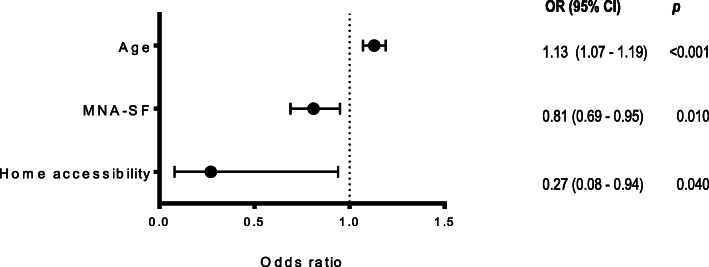
Backward multivariate logistic regression model according to participation in a post-hospitalization physical exercise program. Variables in the first equation included age, walking assistance device, home accessibility, weight, SPPB scores, and Mini Nutritional Assessment Short Form (MNA-SF) metrics. Estimates were based on: *n* = 313 due to missing values; Hosmer-Lemershow goodness of fit, *P* = 0.614; Omnibus *P* < 0.001; and R^2^ Nagelkerke = 0.191

Backward multivariate logistic regression analysis was performed on the variables in the model (age, walking assistance device use, home accessibility, weight, SPPB, and MNA-SF) and revealed that higher age (OR: 1.13; 95% CI: 1.07–1.19), lower MNA-SF test score (OR: 0.81; 95% CI: 0.69–0.95), and lack of home accessibility (OR: 0.27; 95% CI: 0.08–0.94) were independent predictors of participation refusal (Fig. 2).

## Discussion

Difficulty in engaging older people in physical exercise programs is highly prevalent; however, there is limited information about the clinical characteristics that serve as barriers preventing participation in physical post-hospitalization interventions [11]. The present study reveals that older adults have low interest in participating in post-hospitalization physical exercise programs, and that non-participation associates with physical, nutritional, and social parameters. Likewise, increased age, poor nutritional status, and home accessibility problems were strong independent predictors of non-participation.

### Barriers to participation in the exercise program

Most post-hospitalization physical exercise programs are carried out in patients undergoing cardiac or lung rehabilitation or after hip or knee repair. Participation rates reported in these programs (30–70%) are higher than in our program [13, 26]. These differences may be due to physical exercise being considered an essential part of rehabilitation after the above-mentioned treatments. Physical exercise after hospitalization is strongly recommended by the European Society of Cardiology [27], the American Thoracic Society, and the European Respiratory Society [28], and it is usually well accepted by patients, but geriatric patients with broader reasons for hospitalization similar to those in our study (mainly infections) often do not consider exercise a useful intervention after discharge. Higher participation rates were reported in previous post-hospitalization physical exercise programs for older adults; however, the participants in those interventions were slightly younger than in the present study [11, 12]. Nevertheless, the participation rate in our study was similar to other physical exercise programs for community-dwelling older adults, with rates of 7.3–13% in studies with similar participant characteristics [10].

It is remarkable that the main reason for non-participation for half of the patients was a lack of interest in physical exercise. This lack of interest was described in other older adult populations who do not recognize the positive health benefits of physical activity [29, 30]. Other barriers, such as social influences, e.g., lack of encouragement from others, physical limitations (pain or discomfort), or low self-efficacy are present in older populations [31, 32].

In this sense, health professionals may play a fundamental role in informing patients about physical and cognitive deterioration associated with hospitalization [1, 2] and the benefits of physical exercise after discharge to revert these deleterious effects [7]. For these reasons, it is pertinent to actively encourage patients to get involved in physical exercise programs as part of a structured post-hospitalization treatment and take into account patient perceptions and engagement factors when designing exercise interventions for this population.

We also found that poor accessibility at home was an independent factor for non-participation in the physical exercise program. This result agrees with other qualitative studies in which accessibility or environmental barriers hinder access to exercise programs [33]. Usually, poor accessibility is associated with lower socioeconomic status, one of the most important predictors of negative health outcomes [34]. Additionally, transport difficulties were reported as barriers for a quarter of those who declined to participate in the exercise program. This result agrees with other studies that define poor access to transport as a relevant barrier to older people’s physical activity participation [33]. Therefore, special efforts are needed to improve incorporation of people with lower economic status in physical exercise programs and provide economical and accessible transport to facilities where physical exercise is performed.

### Participant characteristics

People who refused to participate were older than those who accepted the exercise program. Logistic regression models showed that the probability of non-participation increased by 13% with each additional year of age. This finding agrees with post-hospitalization cardiac rehabilitation programs where participation declines significantly after 70 years of age, and non-participation rates are even lower at 80 years of age [35]. Similar results were found in lung rehabilitation post-hospitalization programs [36].

We did not find sex-related differences in participation. However, a previous study found significantly lower participation of women after a cardiac event [13]. These differences should be assessed with caution because there may be age, social, or disease-related differences among participants. For instance, older women in the Basque population are as physically active as older men [37], which could account for the lack of sex-related differences in our study.

Additionally, patients who participated in the program had better nutritional status. Interestingly, nutritional status was included in the last equation of the logistic regression model as an independent predictor of participation, which indicates strength of the relationship between nutrition and participation in physical exercise. Malnutrition and poor functional performance are closely interrelated—malnutrition is associated with a higher risk of sarcopenia [38] and, consequently, poorer functional status. Malnutrition in older people is due to multiple factors [39], led by medical illnesses, mental health conditions, psychological causes, or social isolation [40]. Additionally, malnutrition or risk of malnutrition in older people is directly associated with multiple negative outcomes, including increased mortality [41], longer hospital stays, and poorer quality of life [42, 43]. Interventions to improve nutritional and physical status regularly include exercise. Our findings suggest that it is necessary to reduce barriers to developing exercise interventions and to improve older patients’ participation in post-hospitalization exercise programs, especially for patients who generally refuse to participate. Further, lower participation among patients with worse nutritional status is worrying because these individuals are prone to functional deterioration [42].

A strength of this study is its objective clinical measurement of reasons for non-participation in a post-hospitalization physical exercise program and its evaluation of nutritional and functional variables. To our knowledge, few studies have analyzed participation in post-hospitalization physical exercise programs for patient’s receiving care in internal medicine and neurology departments. Further, other studies analyze only qualitative and socioeconomic parameters for older adults’ non-participation [44, 45]. The main limitations of this study relate to it being a secondary analysis of an RCT. On one hand, certain people who would participate in a conventional physical exercise program might not be willing to participate in a research trial. On the other hand, some variables that could be relevant for non-participation have not been assessed, such as self-efficacy, social support, or socio-economic status, which could influence non-participation. In addition, this study is limited by its cross-sectional nature, which excludes any ability to determine temporality and causality. Finally, the fact that there were only 55 participants could reduce the statistical power of the results.

## Conclusions

Participation of older patients in a post-hospitalization physical exercise program was low, especially among patients with poor nutritional status and low accessibility at home. These findings inform strategies to improve accessibility and increase older adult participation in physical exercise programs following hospitalization. To prevent health inequality, non-participants should have the opportunity to receive social support, e.g. assistance with transportation, and nutritional intervention to increase their willingness to participate. Efforts are needed to increase participation in physical exercise programs, specifically in populations that are less prone to participate.

## Data Availability

The datasets used and analyzed during the current study are available from the corresponding author on reasonable request.

## Abbreviations

CC: Calf circumference
MNA-SF: Mini Nutritional Assessment Short Form
MMSE: Mini Mental State Examination
PE: Physical exercise
SPPB: Short Physical Performance Battery
SPMQS: Short Portable Mental Status Questionnaire
